# A trajectory tracking control system for paddle boat in intelligent aquaculture

**DOI:** 10.1371/journal.pone.0290246

**Published:** 2023-08-17

**Authors:** Zhenqi Guo, Junfeng Zhang, Fancong Zeng, Zhijiang Zuo, Libo Pan, Han Li

**Affiliations:** 1 State Key Laboratory of Precision Blasting, Jianghan University, Wuhan, China; 2 School of Intelligent Manufacturing, Jianghan University, Wuhan, China; 3 Wuhan Academy of Agricultural Sciences, Wuhan, China; University of Shanghai for Science and Technology, CHINA

## Abstract

Trajectory tracking plays a notable role in unmanned surface vehicles (USV), especially for the emerging intelligent aquaculture, as the level of integration, high-efficiency, and low-labor-intensity of such USV is determined by trajectory tracking. Here, we report a generic trajectory tracking control system for a paddle boat by establishing a three-degree-of-freedom kinematics model, which could precisely characterize the relationship between velocities, forces and moments of the paddle boat. A Pixhawk 4 as the core controller of the hardware system could be integrated with the other hardware submodules and could complete the wireless data transmission, monitoring and remote control functions. Meanwhile, we establish a fuzzy rule table, consider the advantages of line-of-sight (LOS) guidance and fuzzy adaptive proportional-integral-differential (PID) algorithm, combine the two parts and apply them as the key algorithm in the trajectory tracking of the paddle boat. Demonstrations include trajectory tracking effect at different velocities, turning effect at left-turn moment, and trajectory tracking effect at different turning angles. The results show that the paddle boat is able to travel under the trajectory formed by following the planned waypoints within the error allowed, which is called effective trajectory tracking. And can offer an alternative pathway toward achieving effective trajectory tracking control in advanced intelligent aquaculture USV for smartly and wirelessly operated pond drug spraying.

## 1 Introduction

With the development of modern intelligent technology, unmanned surface vehicles (USV) are increasingly in demand for civilian applications, including environmental protection, water-quality testing and aquaculture, effectively bridging labor shortages and improving production efficiency [1–3]. However, USV have posed some inherent challenges like poor autonomous navigation performance, large trajectory tracking error, poor adaptive ability, and single application scenario that could impact the future of USV market. First, there is too much manual intervention in the application of USV. To reduce labor costs and improve efficiency, it is necessary to combine autonomous navigation and control algorithms to design trajectory tracking. Besides, prescribed route tracking within USV also needs to be carried out as route error may result in yaw and instability control [4, 5]. Currently, an efficient design algorithm is essential for effective navigation performance of USV, and it has been becoming a hot topic of scientific studies in society now. Second, the prompt development of the integration within control system is crucial to the future development of USV. When the control integration is high, the advantages are more obvious, and the adaptive ability can be improved significantly [6]. Lastly, increasing the demand for multi-scene applications of USV is necessary to track more path points, as it leads to more precise of the driving lines and thereby improves the adaptability of USV in different environment.

Hardware structure has been investigated by many researchers, which includes Arduino, PX4, and ARM microcontroller. PX4 is widely adopted because of powerful functionality and open access. Zhao et al. [7] chose Arduino Mega2560 as the controller and built a control system with GPS, a data acquisition module and a motion control module driving four DC motors, which solved the dynamic real-time tracking problem under interference uncertainty and proved the feasibility of this control system. Xiong et al. [8] built Arduino Mega2560, GPS, GPRS, sensors of water quality detection, and motor drive hardware platform to link with a host computer, solving the problem of fixed-point detection for water quality. The literatures [9, 10] selected PX4 as the main controller for the regulation of USV to prove the feasibility of the controller. Following this, we adopted PX4 as the controller, the actuators employed GPS, data transmission module, and drive module as trajectory tracking preparation, including automatic navigation and motion.

The trajectory tracking method has been commonly used to determine the effective control and stable operation. For the appropriate method of the trajectory tracking, it is a prerequisite that the control methodology of the trajectory tracking, either with deterministic model parameters or with uncertain model parameters, are known as precisely as possible. Moreover, the trajectory tracking methods of the control system usually include five types (line-of-sight (LOS) guidance algorithm [11], adaptive proportional-integral-differential (PID) algorithm [12], fuzzy control method [13], sliding mode control method [14], neural network control method [15]) during the regulating of control parameters. These methods could significantly improve control performance. Among them, adaptive PID algorithm, is considered with large amount of calculation, which leads to a cumulative error. Next, although fuzzy control method could correct the error, the accuracy will be reduced when fuzzy processing. So, we apply the control algorithm of adaptive fuzzy PID algorithm in this study, which could lower the error and improve the accuracy at the same time. Furthermore, the sliding mode control method and the neural network control method hardly meet the requirement of practical applications due to computational complexity and inability to accurately characterize the mathematical model. Therefore, to date, LOS is still the most effective and independent of the object under control. Here, we adopted the LOS guidance algorithm because of its advantages such as easy calculation, simple model, and easy trajectory tracking instructions. There have been numerous studies in the last 3–5 years on the LOS proposing diverse solutions of route processing. The literatures [16–18] almost all used the LOS guidance algorithms to address the problem of unstable route travel. Concurrently, many efforts have been paid to design the tracking schemes aiming to calculate position error in real-time. It is no doubt that the precision of heading control determines the effectiveness of tracking. Shen et al. [19, 20] designed an adaptive path following control method for the problem of straight path following for a class of underactuated bio-inspired snake robots on ground facing unknown friction coefficients, and verified the effectiveness of the controller. Cao et al. [21] introduced a homogeneous nonlinear extended state observer and a dynamic surface control in their study, the purpose was to adjust the saturation of the controller. They also compared their findings with other studies. It is noted that the controller could accurately track trajectories in different environment. Setiawan et al. [22] proposed a trajectory planning as the ground station by using PID method. Qiu et al. [23] established a neural network to reduce the computation and employed a hyperbolic tangent function to diminish sliding mode chattering based on an adaptive sliding mode control scheme. Iqbal et al. [24] proposed a fuzzy-immune adaptive system that enhanced the anti-jamming capability of a self-balancing robot system and proves the stability of the system. Moreover, Iqbal et al. [25] proposed a novel adaptive PD-type iterative learning control with robustness and effectiveness in handling nonlinearities. Considering the application characteristics of the fuzzy adaptive PID for heading control, we calculated the heading deviation and continuously adjusted the parameters.

This work aims to develop an accurately-efficient trajectory tracking control system that could be used for on-site baiting, drug spreading and water quality testing in ponds. Additionally, we take a paddle boat, one of USV, as the research object. The control system does not implement a complex operation process but could capture main trajectory characteristics of the paddle boat. To this goal, we designed the LOS guidance and fuzzy adaptive PID algorithm, and achieved autonomous navigation and transmit relevant data such as heading, position and data transmission speed through constructing the hardware and software system. As a result, these data could be monitored in real-time by the host computer. After that, we carried out several tests using a designed operating platform under given routes in the pond to verify the feasibility of the trajectory tracking control. Overall, our trajectory tracking control system may pave the way to realize unprecedented smart aquaculture.

This paper contains 4 sections: in Section 2, the focus is on the methodology. In Section 2.1, a three-degree-of-freedom kinematic model of the paddle boat is built; in Section 2.2, the hardware control device of the trajectory tracking system is constructed; and in Section 2.3, the algorithms and control methods used are highlighted. The experimental results and analysis results are given in Section 3. Finally, some conclusions are presented in Section 4.

## 2 Methodology

### 2.1 Model description

**Fig 1:**
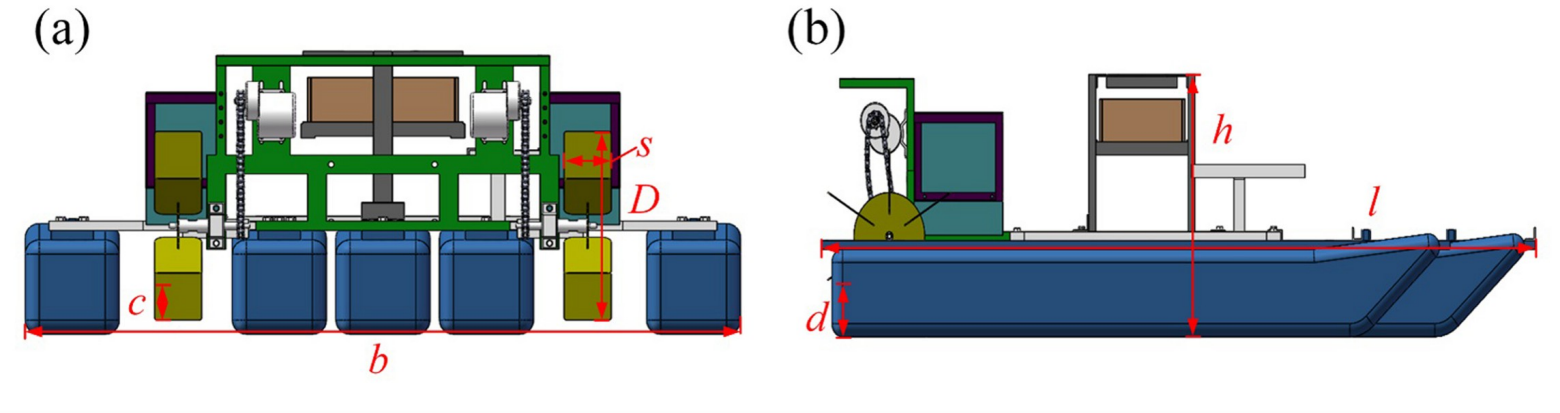
Geometric dimensions of the paddle boat: (a) rear view; (b)side view.

To establish the kinematics model of the paddle boat, dimensions of the geometric structure used in this study are shown in Fig 1. The basic overall dimensions *l*×*b*×*h* equals 1770mm×1600mm×580mm, the water intrusion depths of the boat and two wheels are *d* = 110mm and *c* = 85mm, respectively. Then, the diameter (*D*) and width (*s*) of the wheels are 440mm and 110mm, respectively.

**Fig 2:**
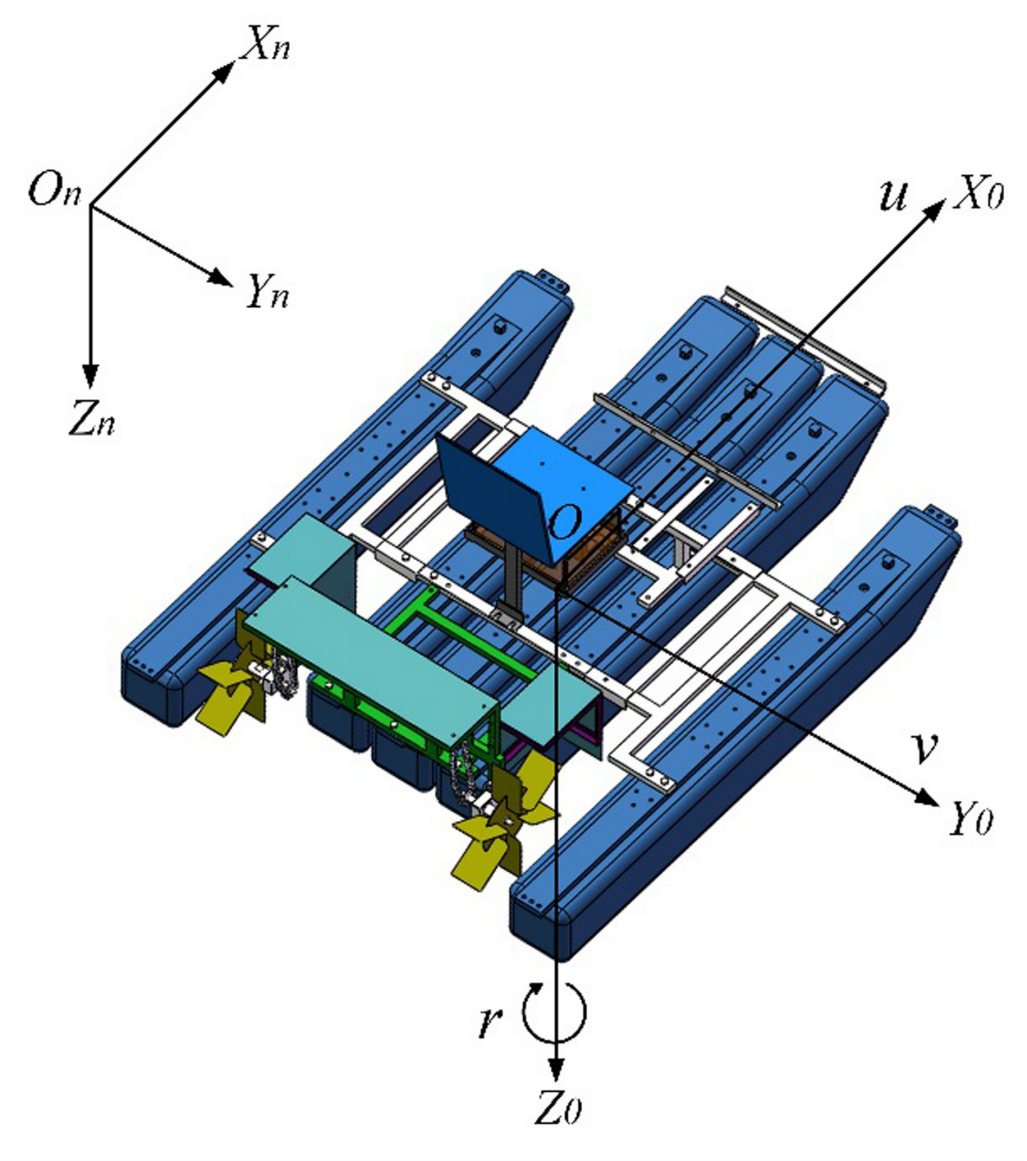
Schematic diagram of inertial coordinate system and hull coordinate system.

It is very important to know the acting force between the paddle boat and water. As a general rule, azimuth, pitch and roll angles have little effect on the motion in the horizontal plane. Therefore, we considered only a simplified kinematic model of that is also proposed by Fossen [26] and was characterized by the ease of observing the acting force, which facilitated analytical calculations. The difference between this model and this paper is that the rudder angle is transformed into the steering angle caused by the speed difference of the paddles. Considering the existing longitudinal swing, transverse swing and bow rocking, based on the inertial coordinate system *O*_*n*_-*X*_*n*_*Y*_*n*_*Z*_*n*_ fixed on the Earth’s surface and hull coordinate system *O*-*X*_*0*_*Y*_*0*_*Z*_0_ fixed on the paddle boat, we set up a three-degree-of-freedom kinematics model, as shown in Fig 2.

The transformation between the inertial coordinate system and the hull coordinate system can be expressed as:

$$\left[\begin{array}{c} \dot{x}\\ \dot{y}\\ \dot{\psi } \end{array}\right]=\left[\begin{array}{ccc} \cos\psi & -\sin\psi & 0\\ \sin\psi & \cos\psi & 0\\ 0 & 0 & 1 \end{array}\right]\left[\begin{array}{c} u\\ v\\ r \end{array}\right]$$ (1)

where, *x*, *y*, and *Ψ* represent the x-point, y-point, and the bow angle of the paddle boat in the inertial coordinate system, respectively; *u*, *v*, and *r* represent the forward velocity, traverse velocity, and the swing angle velocity in the hull coordinate system, respectively.

In solving the simulation model of the paddle boat, we processed the velocities of the left and right wheels as input parameters and the bow angle as an output parameter. Afterward, we analyzed the propulsive forces of the paddle wheels. According to Newton’s second law of motion, the propulsive forces *F*_*L/R*_ generated by the rotations of the left and right wheels in the water are as follows [27]:

$$F_{L/R}=\frac{1}{8}\rho \pi c^{2}sB(\pi n_{L/R}D\cos\theta -V_{0})K_{T}$$ (2)

where, *ρ* is the water density, kg/m^3^; *B* is the number of individual paddle blade; *n*_*L/R*_ is the velocities of the left and right wheels, r/s; *θ* is the angle of blade immersion depth, deg; *V*_*0*_ is the initial velocity, m/s; *K*_*T*_ is the thrust coefficient.

When the rotation speeds of the left and right wheels are the same, the paddle boat travels in a straight line. In contrast, the rotation speeds of the left and right wheels are asynchronous, the left and right thrusts of the paddles could be inconsistent, then a torque will be generated at this time. The torque *N*_*T*_ caused by the rotation speeds difference can be written as:

$$N_{T}=(F_{L}-F_{R})\frac{b}{2}$$ (3)

The torque is controlled by the rudder angle during turning. The correlation between the torque *N*_*T*_ and the virtual rudder angle *δ*_*R*_ of the paddle boat can be expressed as:

$$N_{T}=N_{\delta T}\delta_{R}$$ (4)

where, *N*_*δT*_ is the hydrodynamic coefficient of the rudder angle.

According to the descriptions in literatures [28, 29], the main kinematics variables are the forward traverse velocity *u*, the traverse velocity *v*, and the swing angle velocity *r*, respectively. When the paddle boat is in course holding, trajectory holding or maneuvering below moderate intensity. Consequently, the kinematic equations for the paddle boat are defined as follow:

$$\begin{array}{l} m\dot{u}=X\\ m(\dot{v}+ur+x_{G}\dot{r})=Y\\ I_{Z}\dot{r}+mx_{G}(\dot{v}+ur)=N \end{array}$$ (5)

where, *X* and *Y* are the hydrodynamic forces along the *X*_*0*_-axis and *Y*_*0*_-axis, respectively; *N* is the dynamic moment along the *Z*_*0*_-axis; *m* is the weight of the paddle boat, kg; *I*_*z*_ is the moment of inertia of the hull with respect to the *Z*_*0*_-axis, kg·m^2^; $\dot{u}$ is the navigation acceleration, m/s^2^; $\dot{v}$ is the transverse drift acceleration,m/s^2^;*ṙ* is the bow angle acceleration,°/s^2^; *x*_*G*_ is the coordinate of gravity of the paddle boat, m.

The data from the paddle boat are brought into the hydrodynamic expressions in the literature [26] to obtain the new expressions:

$$\begin{array}{l} Y=Y_{\dot{v}}\dot{v}+Y_{\dot{r}}\dot{r}+Y_{v}v+Y_{r}r\\ N=N_{\dot{v}}\dot{v}+N_{\dot{r}}\dot{r}+N_{v}v+N_{r}r+N_{\delta T}\delta_{R} \end{array}$$ (6)

where, $Y_{\dot{v}},Y_{\dot{r}},Y_{v},Y_{r},N_{\dot{v}},N_{\dot{r}},N_{v},N_{r}$ are the hydrodynamic coefficients.

Ignoring the viscous force of water, Eq (5) represents the inertia forces and moments along *X*_*0*_, *Y*_*0*_ and *Z*_*0*_-axis. Usually, the forward motion in the *X*_*0*_-axis direction is independent of the motion in the other two-degree-of-freedom. In addition, from the perspective of velocity control, the motion on the *X*_*0*_-direction degree-of-freedom can be considered separately, while there is a strong coupling between the traverse and the swing, which are closely related to the heading and trajectory control of the paddle boat. Combined with Eq (6), the expression for the paddle boat can be written as:

$$\left[\begin{array}{cc} m-Y_{\dot{v}} & mx_{G}-Y_{\dot{r}}\\ mx_{G}-N_{\dot{v}} & I_{Z}-N_{\dot{r}} \end{array}\right]\left[\begin{array}{c} \dot{v}\\ \dot{r} \end{array}\right]+\left[\begin{array}{cc} -Y_{v} & mu-Y_{r}\\ -N_{v} & mx_{G}u-N_{r} \end{array}\right]\left[\begin{array}{c} v\\ r \end{array}\right]=\left[\begin{array}{c} 0\\ N_{\delta T} \end{array}\right]\delta_{R}$$ (7)

The conversion of the state-space model into a linear response model expressed as a transfer function has been widely used in the design of the heading controllers of USV. The paddle boat moves with low-frequency properties and adopts the Nomoto first-order model (Nomoto) on the basis of neglecting the lateral drift velocity, which facilitates the simulation calculation of the motion. Thus, the transfer function is [30]:

$$G{\left(s\right)}_{\delta R}=\frac{K}{s(Ts+1)}$$ (8)

where, *K* and *T* are the manoeuvring coefficients of the paddle boat.

### 2.2 Hardware aspect and working process

**Fig 3:**
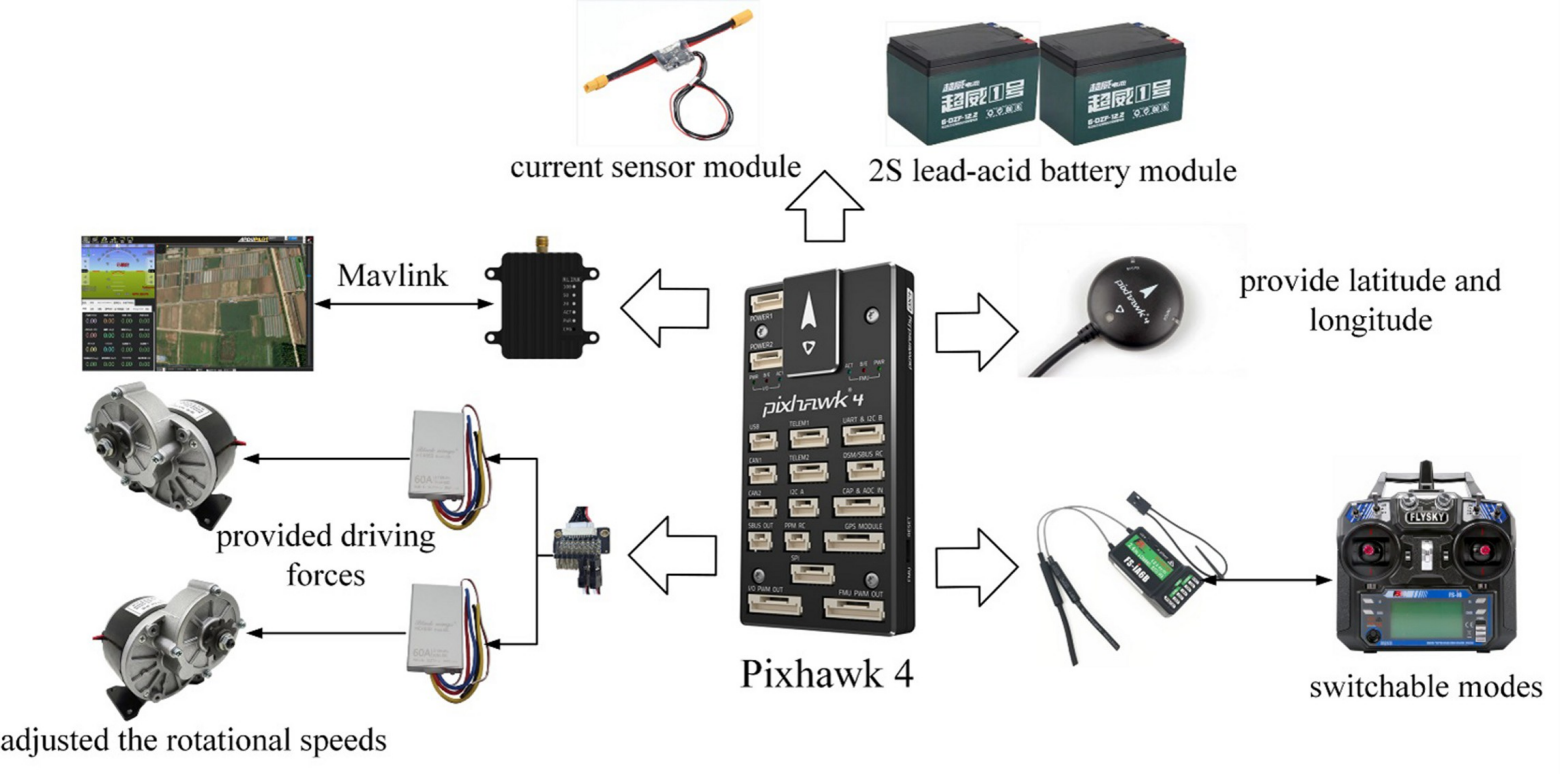
Hardware control system.

This work implemented a series of experimental testings for validation using the hardware control system shown in Fig 3. It shows the overall component hardware, and the experimental modules include a main controller, a power supply system, a communication system, a positioning system, and a driving system. The paddle boat was able to accomplish the mission of autonomous navigation with the cooperation of various subsystems. These experiments employed a main controller (Pixhawk 4) to generate well-controlled autonomous sailing to study the trajectory tracking of the paddle boat. The power supply system consist of a 2S (S stands for cell connection in series) lead-acid battery module and a current sensor module. In fact, the battery module was capable of generating power for the main controller, GPS, remote control, receiver and other devices. In addition, the main functions of the current sensor module were: (i) monitored the values of the current and voltage. (ii) dropped and regulated the voltage.

The communication system used include a receiver, a remote control to operate and switch modes, a data transmission module, and a host computer to connect with the computer software through the Mavlink protocol (mainly used for the unmanned carrier communication). The working process of the communication system was described as follows:

- We built a connection between the ground side and the control side by using the data transmission module, which could collect 50 data per second, resulting in a high data collection effort. Therefore, we chose a mean filtering algorithm in the data acquisition, and extracted the continuous sampled data per second for accumulation, then assigned the obtained mean value to the data at this time. This type of processing could guarantee the stability of the output data. The formula about the assignment data is given by:

$$b=\frac{\sum_{i=1}^{n}b_{i}}{n},\;i=1,2,3\ldots n$$ (9)

where, *b*_*i*_ is the i-th sample, n is the number. Then, the host computer sent out the information of the waypoints, position, and velocity, and monitored the track.

- We designed three main navigation modes, namely hold mode, automatic mode, and manual mode. The different modes could be obtained by switching the host computer or remote control, where corresponded to pulse width modulation signals 0 to1230, 1231 to1360, and 1361 to 1490, respectively.
- We compiled control algorithms to solve for waypoints and positions, and calculated the course and position that the paddle boat would travel.

The core of a positioning system is GPS, which is used to find the exact location of the paddle boat by longitude and latitude. A driving system consist two components: two electronic governors and two motors, they provide driving forces and adjusted the rotational speeds for the left and right wheels, respectively. The process of the speed control could be described as follows: first, to calibrate the motors, we connected the signal lines of the electronic governors to the receiver; second, we linked the signal lines to the pulse position modulation decoder board, aiming to receive the motion command signal and spin the motors.

All components were assembled together using the hardware control system, the details of which are shown in Table 1.

**Table 1:** Hardware model selection.

Equipment	Model	Performance
Main controller	Pixhawk 4	/
Processor	STM32F765	Cortex M7-512KB RAM
Power supply	2S lead-acid battery module	24V
Receiver	FS-iA6B	Receiving distance 500 to 1500m
Remote control	FS-i6	Remote distance 500 to 1500m
Data transmission module	Made by blicube from China	Stable distance 20km
GPS	M8N	Positioning accuracy ±1500mm
Electronic governor	HC4860	/
Motor	MY1016Z3	Rated speed 3000rpm

### 2.3 Algorithms and control

**Fig 4:**
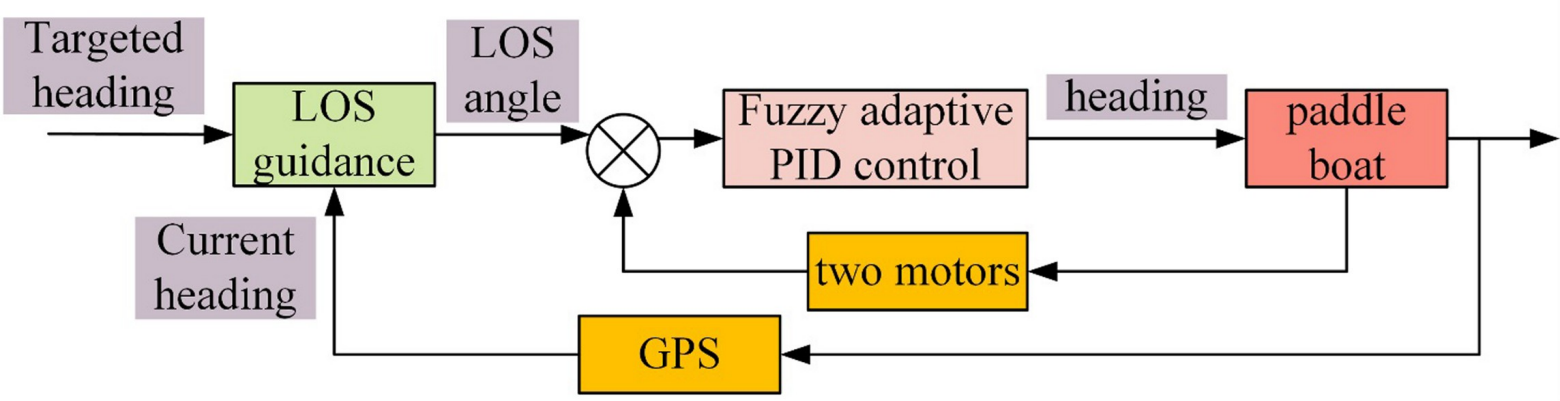
Control strategy of the trajectory tracking.

As stated in section 2.1, the velocity and turning of the paddle boat are adjusted by the rotational speed and rotational speed difference between the left and right wheels, respectively. At this point, the turning strategy is to rotate one motor and stop the other one. Additionally, to enable the paddle boat to navigate under the prescribed route, we further develop the trajectory tracking control system in this study, which is mainly composed by two parts, namely LOS guidance system and fuzzy adaptive PID control system, and Fig 4 shows the diagram of the control strategy.

**Fig 5:**
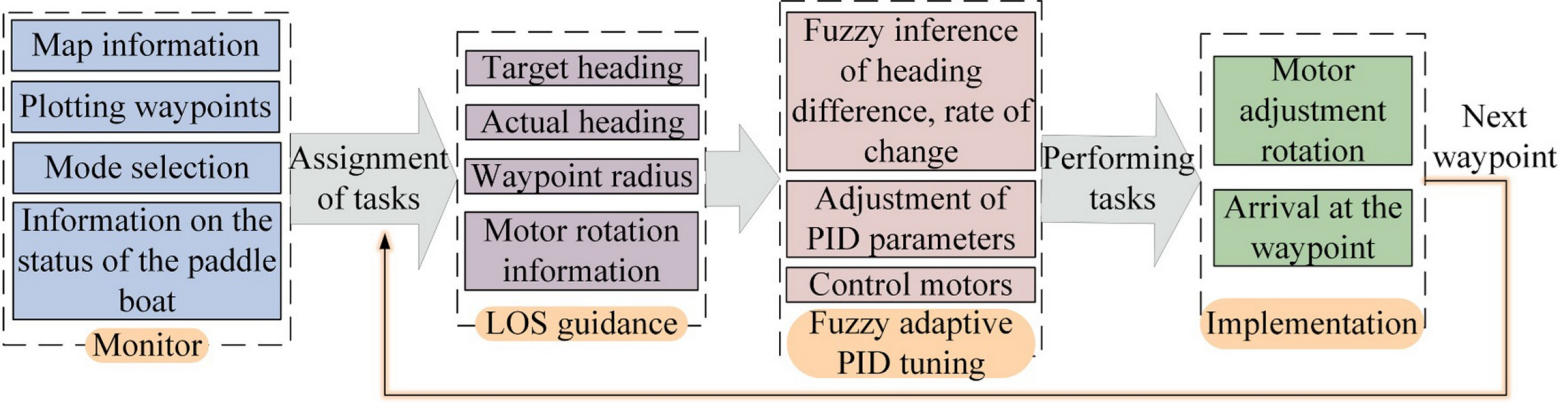
Control operation flow.

The trajectory tracking control operation includes the host computer monitoring system, the tracking control system and the execution system, where the tracking control system includes the LOS guidance and the fuzzy adaptive PID control algorithms, as shown in Fig 5. The host computer displays the current heading and tracks the target heading, and the two motors make the relevant commands after being solved by the previously mentioned control algorithm.

#### 2.3.1 LOS implementation

The LOS guidance algorithm is able to select line-of-sight points on this section of the track formed by the starting point and the target point when the paddle boat is underway, and controls the course of the paddle boat to navigate in the desired direction.

**Fig 6:**
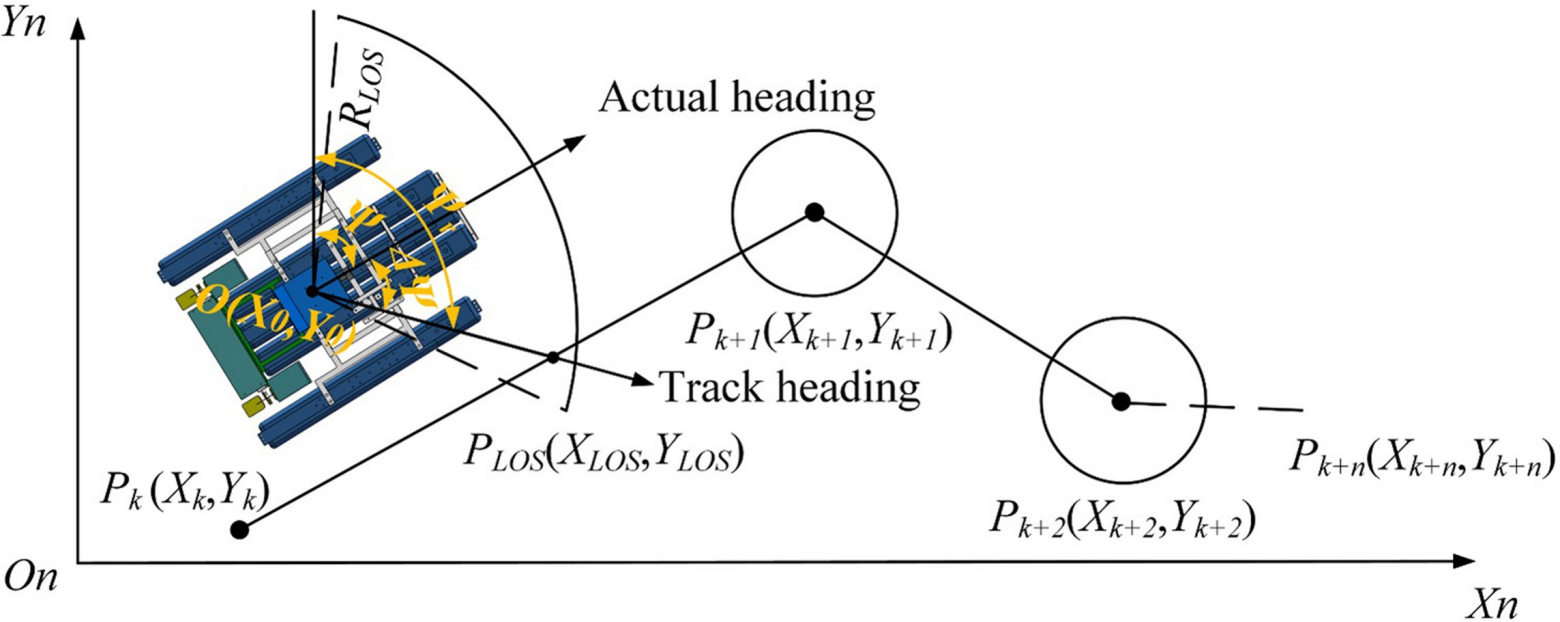
LOS guidance principle.

We set the starting point at *P*_*k*_ (*X*_*k*_,*Y*_*k*_) and the target point *P*_*k+1*_(*X*_*k+1*_,*Y*_*k+1*_) in the inertial coordinate system *O*_*n*_-*X*_*n*_*Y*_*n*_*Z*_*n*_, and straight line *P*_*k*_*P*_*k+1*_ was the preset trajectory of the paddle boat, as illustrated in Fig 6. The real-time position of the paddle boat can be expressed as:

$${\left(X_{LOS}-X_{0}\right)}^{2}+{\left(Y_{LOS}-Y_{0}\right)}^{2}=R_{LOS}^{2}$$

$$\frac{Y_{LOS}-Y_{k}}{X_{LOS}-X_{k}}=\frac{Y_{k+1}-Y_{k}}{X_{k+1}-X_{k}}$$ (10)

where, *X*_*0*_ and *Y*_*0*_ are the horizontal and vertical coordinates of the initial position of the paddle boat, respectively; *R*_*LOS*_ is the sailing radius, which is a variable, *R*_*LOS*_ = *nl*, and n is the captain ratio. From Fig 6, it could be seen that when the turning radius was set to the variable *R*_*LOS*_, the left and right limit radii could form a sector region. Then we marked *P*_*LOS*_(*X*_*LOS*_,*Y*_*LOS*_) as the intersection of this sector region with the preset trajectory.Assuming that the positive direction of the *Y*_*n*_-axis is north, then we record the target heading angle in real-time between the north direction and the straight line *OP*_*LOS*_ as *Ψ*_*r*_, and *Ψ*_*r*_ can be written as:

$$\psi_{r}=\arctan(\frac{Y_{LOS}-Y_{0}}{X_{LOS}-X_{0}})$$ (11)

At the same time, we used GPS positioning system to determine the actual heading angle *Ψ*. Then by calculation we got the deviation angle *ΔΨ*, which was the difference between *Ψ*_*r*_ and *Ψ*. We could also observe from Fig 6 that when the paddle boat entered the circular area at point *P*_*k+1*_, the target point would be shifted to *P*_*k+2*_ and at this time, *P*_*k+1*_ would be associated with *P*_*k+2*_ by iteration. In this way, the calculation continued in this iterative manner until the computation reached the point *P*_*k+n*_ point. Based on the above LOS algorithm analysis, it was understandable that the position of the *P*_*LOS*_ point could be deduced from the real-time position, the starting point and the target point of the paddle boat [31].

#### 2.3.2 Fuzzy adaptive PID control system

PID control algorithm is a control algorithm that combines proportional, integral and differential links in one, which can be expressed as a function of the deviation as [32]:

$$u(t)=K_{p\text{i}}[e(t)+\frac{1}{T_{i}}\int_{0}^{t}e(t)dt+T_{d}\frac{de(t)}{dt}]$$ (12)

where, *u(t)* represents the control output; *e(t)* represents the deviation between the actual heading angle and the target heading angle; *K*_*pi*_ represents the proportional gain; *T*_*i*_ and *T*_*d*_ represent the integration time constant and the differential time constant, respectively.

It is no doubt that the three parts of PID, proportional, integral and differential, are linear, thus resulting in a PID controller being a linear controller. However, the paddle boat exhibits a nonlinear characteristic during navigation. If we apply the linear PID controller to the trajectory tracking of the paddle boat, it may reduce the regulation accuracy. At the same time, it needs to adjust the parameters artificially continuously during the control process, which may not applicable to practical situations. Compared with general PID linear controller, the fuzzy adaptive PID control has a better nonlinear characteristic, a higher stability and reliability, can achieve system stability in the fastest time and the least amount of overshoot. Therefore, it is necessary to combine the experience of manual operation with the fuzzy control on the basis of general PID, thus forming the fuzzy PID control algorithm, which can yield more precise results.

**Fig 7:**
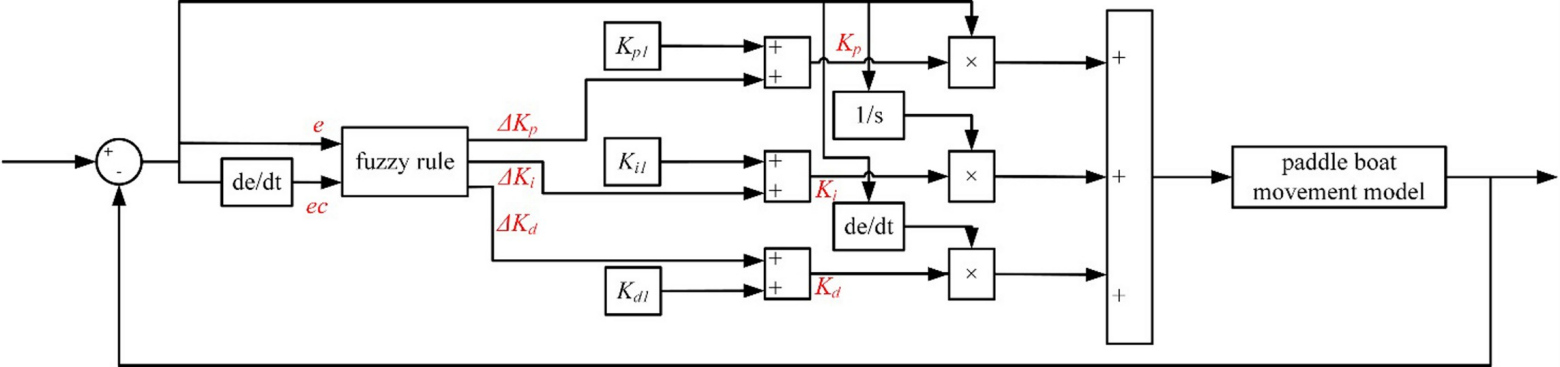
Fuzzy adaptive PID control process.

The fuzzy controller consists of three main parts: the fuzzy input, the fuzzy inference rule, and the fuzzy output. The inputs we set were e and ec, which were the heading error between the given heading and the actual heading as well as the heading error rate, respectively. Additionally, we used the adjusted values of the PID parameters *K*_*p1*_, *K*_*i1*_, and *K*_*d1*_, i.e. *ΔK*_*p*_, *ΔK*_*i*_, and *ΔK*_*d*_, as outputs. We summed the *ΔK*_p_, *ΔK*_*i*_, and *ΔK*_*d*_ of the fuzzy control output with *K*_*p1*_, *K*_*i1*_ and *K*_*d1*_ of the PID, respectively, to obtain the adjusted *K*_*p*_, *K*_*i*_, and *K*_*d*_, as shown in Eq (13) and Fig 7.

$$\begin{array}{l} K_{p}=K_{p1}+\Delta K_{p}\\ K_{i}=K_{i1}+\Delta K_{i}\\ K_{d}=K_{d1}+\Delta K_{d} \end{array}$$ (13)

where, *K*_*p1*_, *K*_*i1*_, and *K*_*d1*_ are the proportional, integral and differential coefficients of the PID, respectively, which are constantly adjusted by the experimental process of referencing; *ΔK*_*p*_, *ΔK*_*i*_, and *ΔK*_*d*_ are the proportional, integral and differential coefficients of the fuzzy control, respectively; *K*_*p*_, *K*_*i*_, and *K*_*d*_ are the proportional, integral and differential coefficients of the final action on the paddle boat, respectively.

In the fuzzy adaptive PID control, we designed the fuzzy inference rules using a 7-segment fuzzy method, setting the theoretical domain ranges of both e and ec to [-6, 6], *ΔK*_*p*_, *ΔK*_*i*_, and *ΔK*_*d*_ to [-6, 6], [-0.6, 0.6], and [-10, 10], respectively. And, we set the subsets of the fuzzy to {NB, NM, NS, ZO, PS, PM, PB}, the element within the subsets represents {negative large, negative medium, negative small, zero, positive small, positive medium, positive large}. The main rules of the regulation for each period in fuzzy adaptive PID were: (i) in the early stage, the proportional coefficient of the fuzzy control *ΔK*_*p*_ should be taken as a larger value to improve the response speed. At the same time, to prevent the integral saturation, the integral coefficient *ΔK*_*i*_ should be as small as possible, or even can take zero. In addition, the differential coefficient *ΔK*_*d*_ should be taken as a small value to avoid overshooting. (ii) in the mid-regulation, a smaller *ΔK*_*p*_ should be taken in order to make the overshoot of the system smaller and ensure a high response rate. Thus, to avoid affecting the stability of the system, *ΔK*_*i*_ was taken as an intermediate value. Since the regulation characteristics are sensitive to the change of *ΔK*_*d*_ value, the *ΔK*_*d*_ value should be appropriately smaller and should be kept constant. (iii) in the post-regulation, larger *ΔK*_*p*_, *ΔK*_*i*_ and smaller *ΔK*_*d*_ were chosen to reduce the static error, improve the control accuracy and reduce braking effects of the controlled process, respectively. We chose the input and output variables as triangular affiliation functions and determined the corresponding fuzzy rules using the center of gravity method, the details of which are shown in Table 2.

**Table 2:** Fuzzy rules for *ΔK*_*p*_, *ΔK*_*i*_, and *ΔK*_*d*_.

***ΔK***_***p***_ **/*ΔK***_***i***_ **/*ΔK***_***d***_			**ec**						
			NB	NM	NS	ZO	PS	PM	PB
**e**	NB	PB/NB/PS		PB/NB/PS	PM/NM/NB	PM/NM/NB	PS/NS/NB	PS/ZO/NM	ZO/ZO/ZO
	NM	PB/NB/PS		PM/NB/NS	PM/NM/NB	PS/NS/NM	PS/NS/NM	ZO/ZO/NS	NS/ZO/ZO
	NS	PM/NB/ZO		PM/NM/NM	PS/NS/NM	PS/NS/NM	ZO/NS/NS	NS/PS/NS	NS/PS/PS
	ZO	PM/NM/ZO		PS/NM/NM	PS/NS/NS	ZO/ZO/NS	NS/ZO/NS	NM/PM/NS	NM/PM/PM
	PS	PS/NM/ZO		PS/NS/NS	ZO/ZO/ZO	NS/PS/ZO	NS/PS/ZO	NM/PM/ZO	NM/PB/PB
	PM	ZO/ZO/PB		ZO/ZO/NS	NS/PS/PS	NM/PS/PS	NM/PS/PS	NM/PB/PB	NB/PB/PB
	PB	ZO/ZO/PB		NS/ZO/ZO	NS/PS/PM	NM/PM/PS	NM/PM/PS	NS/PB/PB	NB/PB/PB

## 3 Results and discussion

**Fig 8:**
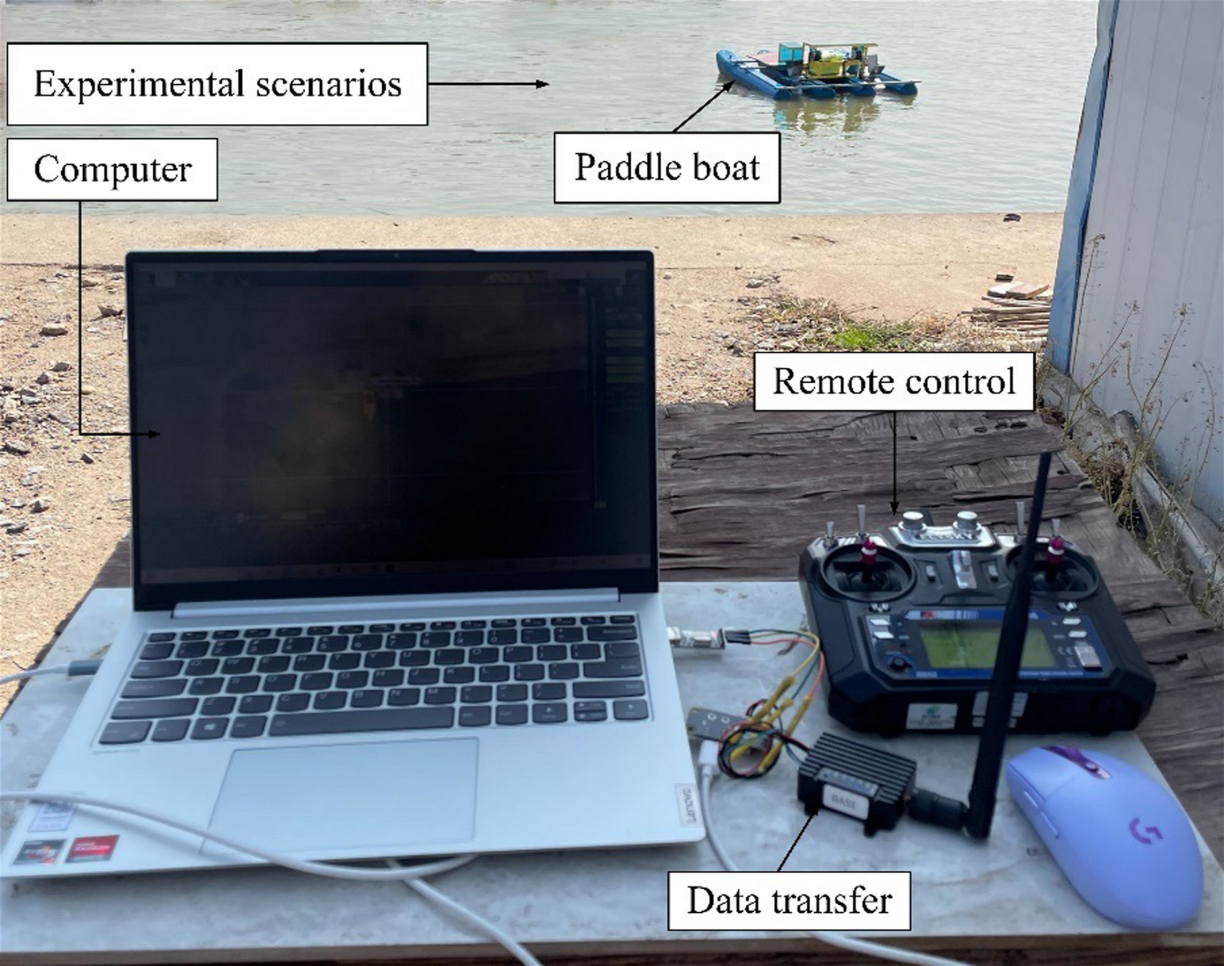
Test operating platform.

Fig 8 shows a test operating platform. We tested the trajectory tracking of the paddle boat in a pond, with a size of 9,000mm×100,000mm, and the weather on the day of the test was sunny, with the temperature ranging from 10 to 25°C and wind force 2 to 3. According to the software and hardware platform built in the previous section, we also created the host monitoring for the Mission Planner platform, a ground station software of open access developed in C#. Under these conditions, we set the radius of the waypoint to 2,000mm and the visible radius of the LOS guidance algorithm to 3,000mm. To further verify the applicability of the trajectory tracking control system, we carried out the tests in two ways. One was to compare the trajectory tracking at different velocities on the same route to select the optimal velocity, the other was to compare different turning angles under various preset trajectories to observe the effect of the trajectory tracking.

### 3.1 Trajectories at different velocities

**Fig 9:**
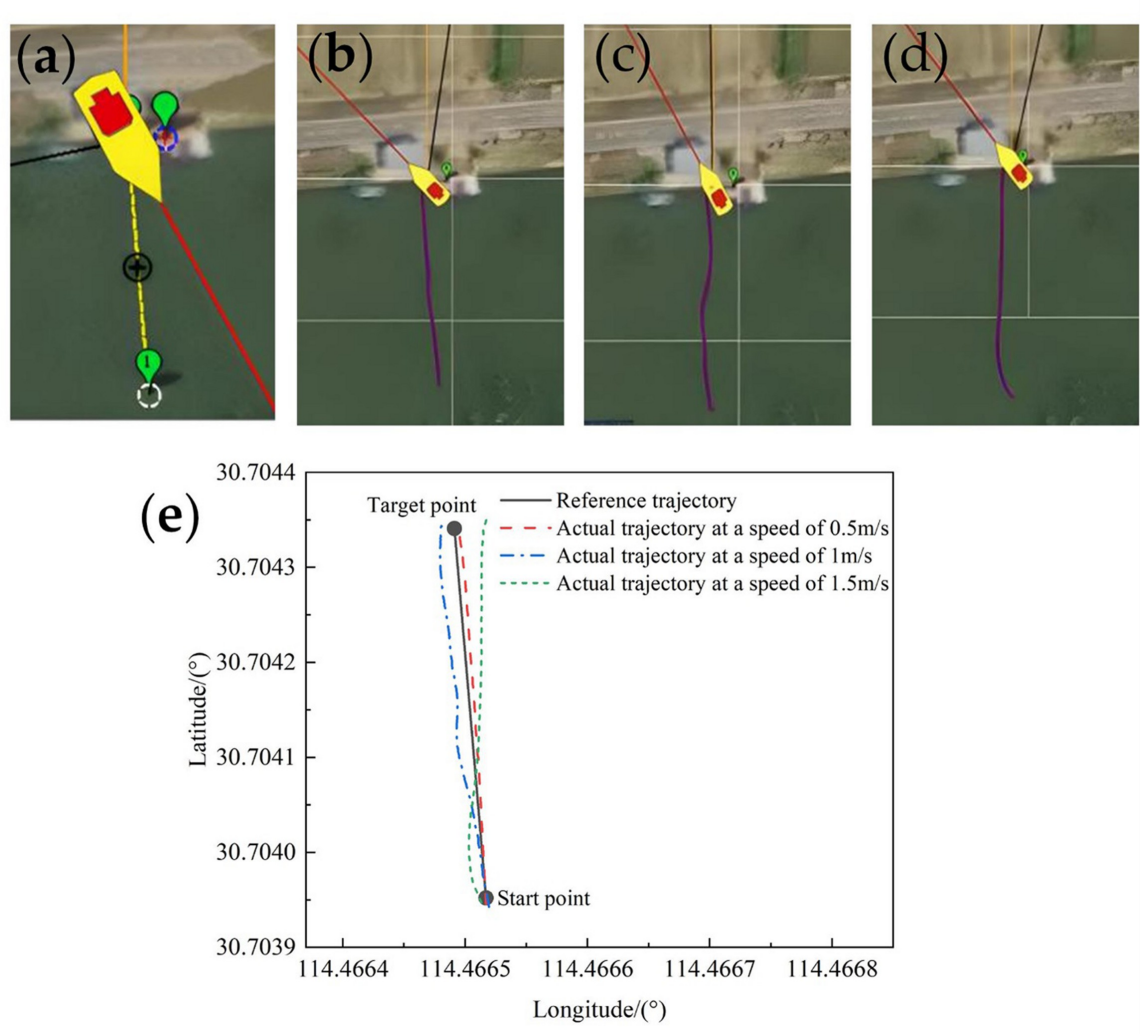
Comparison of trajectory tracking at different velocities on the same route:(a) A straight line reference trajectory; (b) Actual trajectory at a speed of 0.5m/s; (c) Actual trajectory at a speed of 1m/s; (d) Actual trajectory at a speed of 1.5m/s; (e) Comparison between the reference trajectory and the actual tracks.

As you know, when the velocity of the paddle boat is relatively fast, it is easy to be disturbed by wind and waves, even cause deviation of the course. In contrast, when the velocity of the paddle boat is relatively slow, it may affect the efficiency of the work. Therefore, we selected the velocities of 0.5m/s, 1m/s and 1.5m/s for the comparison tests of the trajectory tracking on the same route. Fig 9 shows the comparison of the trajectory tracking under different velocities. In Fig 9(A), the position of the paddle boat was the origin, i.e. the starting point. The green marker indicated the first few target points, the red line was the current heading of the paddle boat, the black line was the GPS tracking line, and the orange line expressed the next waypoint index.

As shown in Fig 9, the overall trend of trajectory were consistent obviously within the error allowed. Apparently, when the velocity increased, the tracking error also increased gradually. However, we do not consider the effect of wind and waves on the heading in our algorithm, increasing boat velocity will lead to the adaptive PID control algorithm to fail to keep up with the convergence rate. And yet, when the heading error e between the given heading and the actual heading reached the set value, the LOS guidance algorithm could assist in turning and correcting the deflection to make the paddle boat towards the target waypoint, proving the stability of this control system. By comparing the cases above, it can be seen that the actual trajectory of the paddle boat at 0.5m/s matches better with the reference trajectory. Therefore, 0.5m/s was chosen for the subsequent tests.

### 3.2 Trajectories at different turning angles

**Fig 10:**
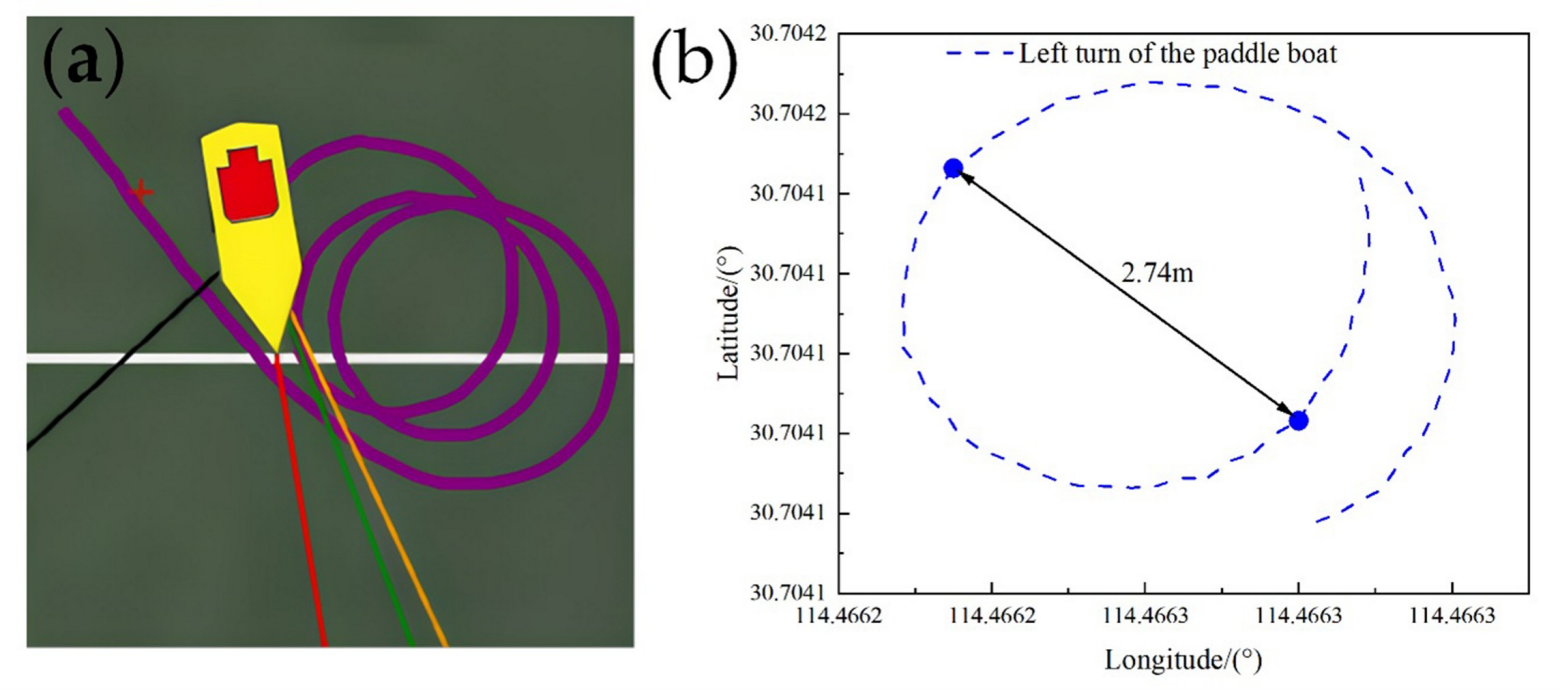
Left turn of the experimental boat: (a) Actual trajectory when testing turning radius; (b) Extracted left-turn trajectory.

**Fig 11:**
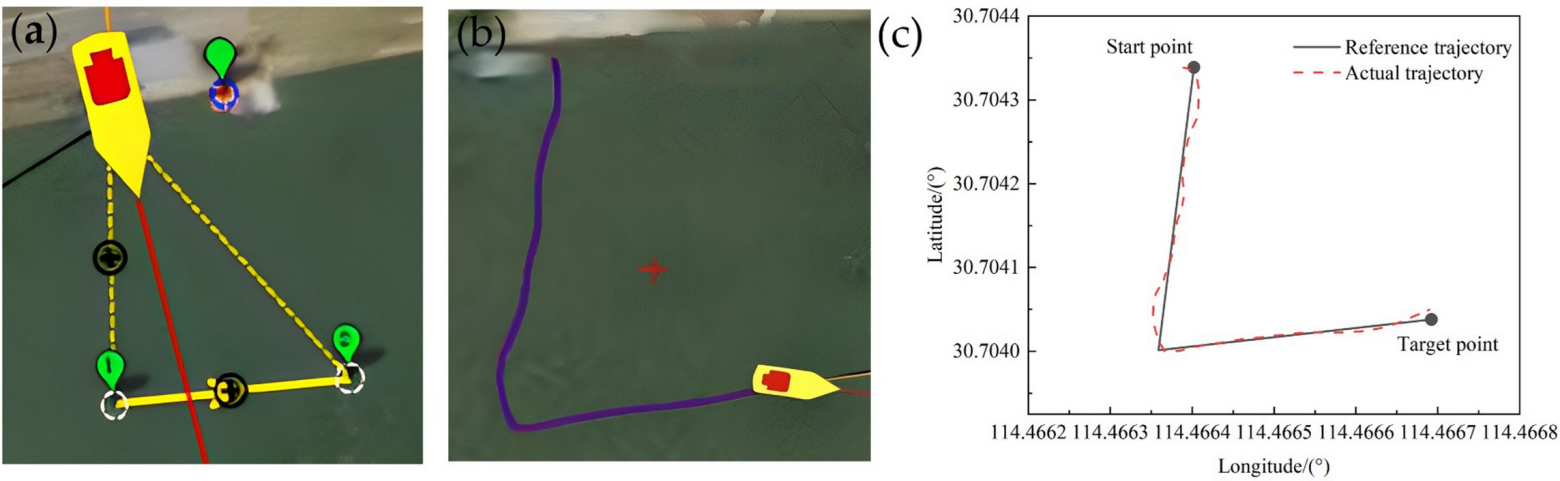
Trajectory of a triangle with an approximate 60° turning angle: (a) Reference trajectory; (b) Actual trajectory; (c) Comparison of the reference trajectory and the actual trajectory.

**Fig 12:**
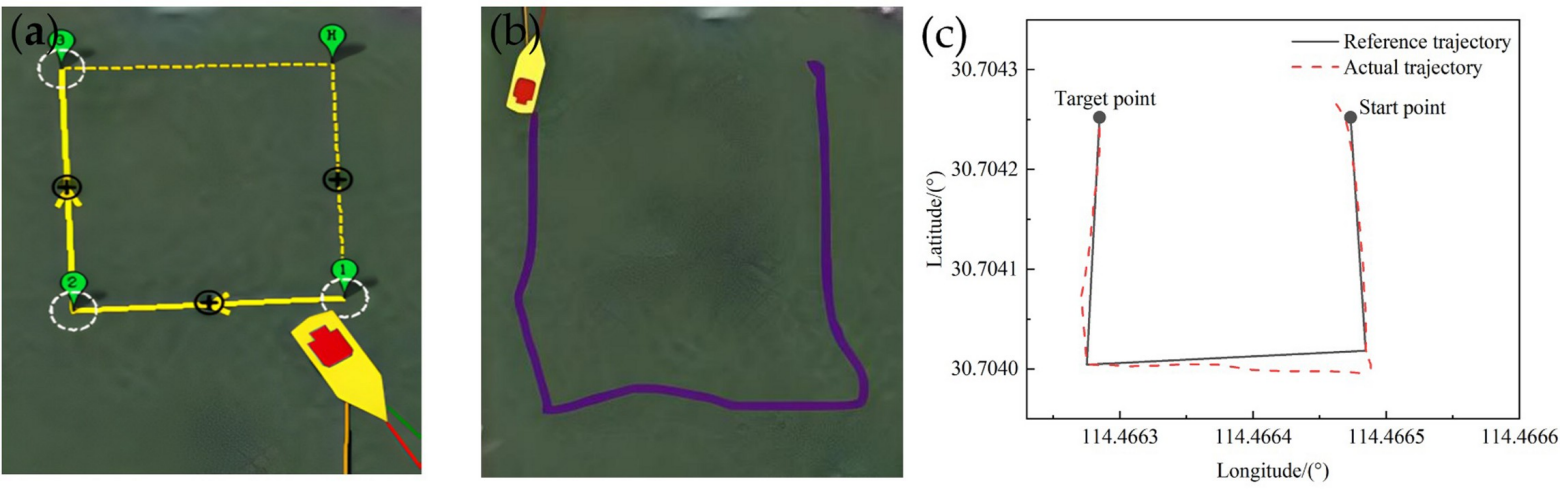
Trajectory of a square with an approximate 90° turning angle: (a) Reference trajectory; (b) Actual trajectory; (c) Comparison of the reference trajectory and the actual trajectory.

**Fig 13:**
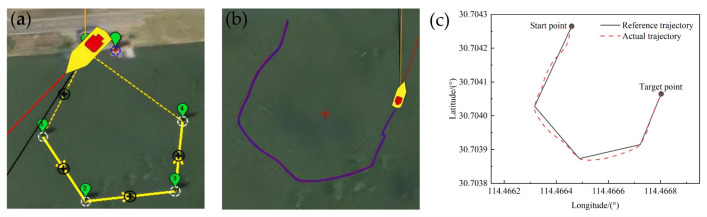
Trajectory of a pentagon with an approximate 120° turning angle: (a) Reference trajectory; (b) Actual trajectory; (c) Comparison of the reference trajectory and the actual trajectory.

In the actual application scenario, the paddle boat is required to adapt to diverse shapes and areas of ponds, resulting in accurate tracking under different turning angles. To be able to perform tasks in different scenarios, it is necessary to verify the turning angle for the accurate trajectory tracking, see Fig 10.Through the actual measurement we found that the small boat turning was very flexible and its turning radius was about 2 m. Here, we set the turning angles as acute (about 30°), right angle (about 90°) and obtuse angle (about 120°), respectively, and studied the tracking of the reference trajectory by the paddle boat when the reference routes were triangle, square and pentagon, respectively. The test results were shown in Figs 11–13.

From the Figs 11–13, it could be observed that with the LOS guidance algorithm and fuzzy adaptive PID control system, the paddle boat could achieve the trajectory tracking under different turning angles with a fixed velocity of 0.5m/s. By comparing the trajectory tracking errors with those under different literatures, it is found that the average error in this paper tracks the best and the maximum error is relatively good, as shown in Table 3. We expand on this with pentagon trajectory tracking and make an experimental analysis of trajectory tracking under different shapes. The actual trajectory and the reference trajectory were in good agreement, but had slightly difference at different turning angles. The reasons are mainly the following two parts: (1) the distance between points *P*_*k*_ and *P*_*K+1*_ may be shorter than the turning radius. Therefore, even if the minimum turning radius is maintained, the actual trajectory may still deviate from the reference trajectory. (2) the fluid and wind in the actual test will affect the experimental results and thus lead to deviation from the reference trajectory. However, when the distance is longer than the turning radius, the LOS guidance algorithm will automatically solve the next tracking point and use fuzzy adaptive PID control to correct the heading, and thereby to achieve trajectory tracking. Overall, the navigation error is within the allowed range, which proves that the control system has high reliability.

**Table 3:** Comparison of trajectory tracking under different literatures.

Literature	Triangle path		Square Path	
	Maximum error	Average error	Maximum error	Average error
Literature [33]	3.6m	1.8m	3.8m	1.5m
Literature [8]	2.7m	1.53m	2.4m	1.89m
Present work	2.48m	1.03m	2.63m	1.26m

## 4 Conclusions

The present paper reports a trajectory tracking control system for a paddle boat. In addition, we successfully develops a test operating platform based on Mission Planner for monitoring navigation track in the paddle boat and providing control method recommendations using the LOS guidance and fuzzy adaptive PID algorithm. We also test the trajectories at different velocities and turning angles successfully using the software and hardware control system and evaluate using preciseness. The significant observations based on the present investigation are listed below:

- A three-degree-of-freedom kinematics model is set up to study the navigation of the paddle boat in the water. Moreover, the simplified model can describe the relationship between the velocities, forces and moments of the paddle boat.
- The overall hardware includes a main controller, a power supply system, a communication system, a positioning system, and a driving system. All components are assembled together and work with each other to perform the task of autonomous navigation.
- The LOS guidance algorithm is used to calculate the LOS angle of the preset track, and the waypoint is tracked and analyzed. Simultaneously, the heading is controlled by fuzzy adaptive PID, and the parameters of the PID are constantly corrected through the transformation of the paddle boat’s heading to make the trajectory tracking more accurate.
- By testing the trajectory tracking effect of different velocities and turning angles under the same route, it is found that the system has good tracking effect under 0.5m/s, and can execute each waypoint stably according to the preset trajectory with good control performance. In addition, the paddle boat in this study has a good turning effect.

Furthermore, these results are expected to provide some significant physical insights for the design and trajectory tracking optimization of such paddle boat. As a perspective for this work, the disturbance of the system by noise such as wind, fluid velocity and water waves cannot be neglected and there is a strong coupling with the motion of the paddle boat, such system will be investigated and analyzed in the near future based on the developed model.

## Supporting information

S1 Data (XLSX)

S1 Raw images (PDF)
