# Supplementary material for: A trajectory tracking control system for paddle boat in intelligent aquaculture
Source: PLoS One. 2023 Aug 17;18(8):e0290246. doi: 10.1371/journal.pone.0290246 (PMC10434876; doi:10.1371/journal.pone.0290246)
Supplement: S1 Raw images — (PDF) [file pone.0290246.s002.pdf]

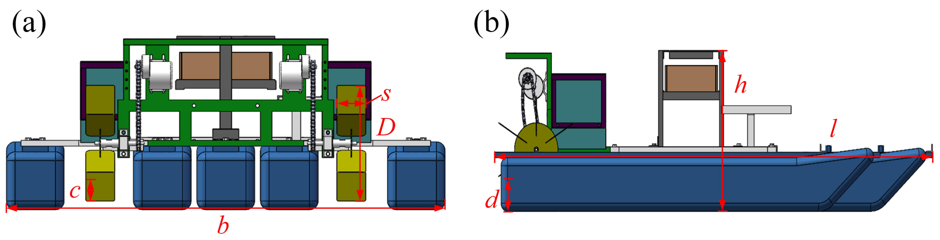

Fig 1. Geometric dimensions of the paddle boat: (a) rear view; (b)side view.

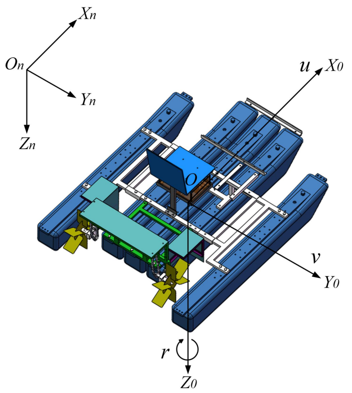

Fig 2. Schematic diagram of inertial coordinate system and hull coordinate system.

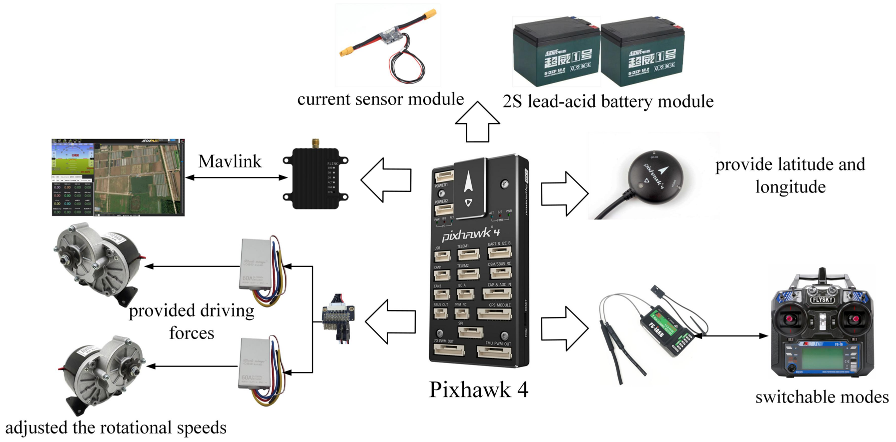

Fig 3. Hardware control system.

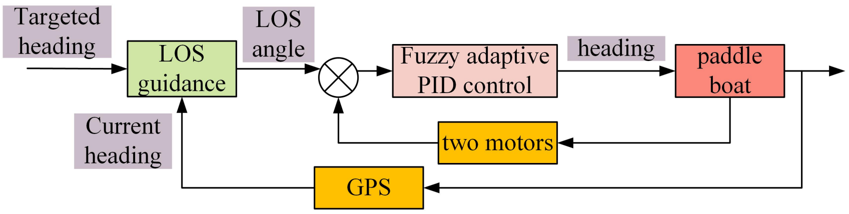

Fig 4. Control strategy of the trajectory tracking.

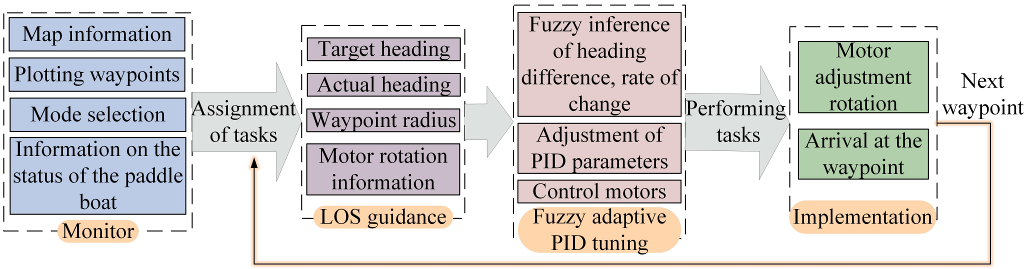

Fig 5. Control operation flow.

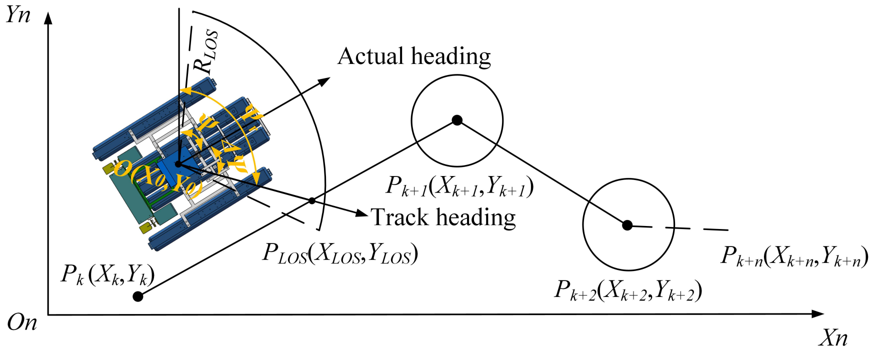

Fig 6. LOS guidance principle.

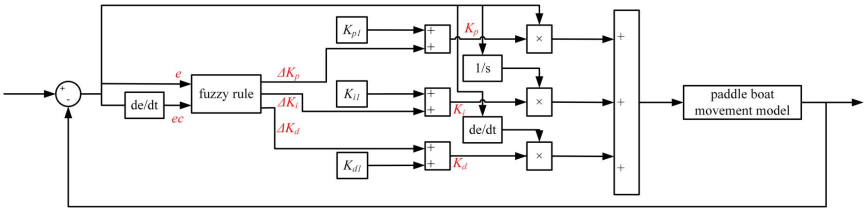

Fig 7. Fuzzy adaptive PID control process.

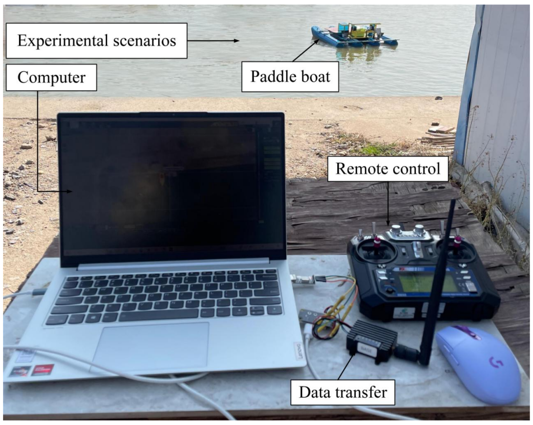

Fig 8. Test operating platform.

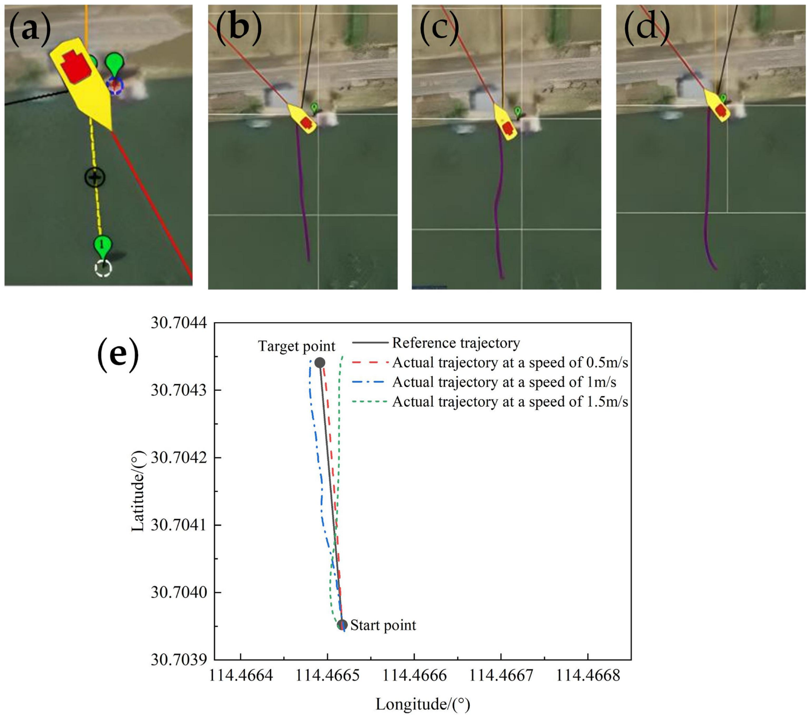

Fig 9. Comparison of trajectory tracking at different velocities on the same route:(a) A straight line reference trajectory; (b) Actual trajectory at a speed of 0.5m/s; (c) Actual trajectory at a speed of 1m/s; (d) Actual trajectory at a speed of 1.5m/s; (e) Comparison between the reference trajectory and the actual tracks.

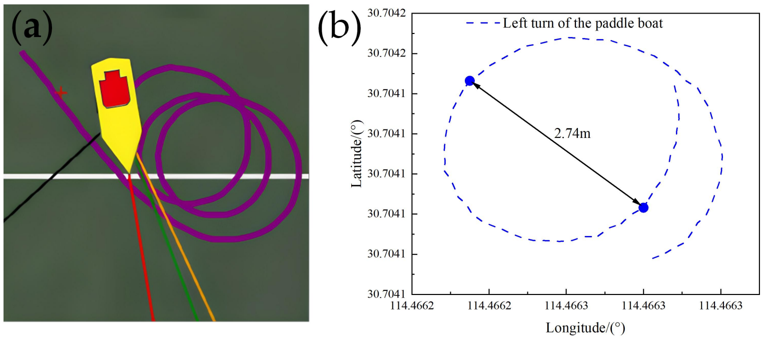

Fig 10. Left turn of the experimental boat: (a) Actual trajectory when testing turning radius; (b) Extracted left-turn trajectory.

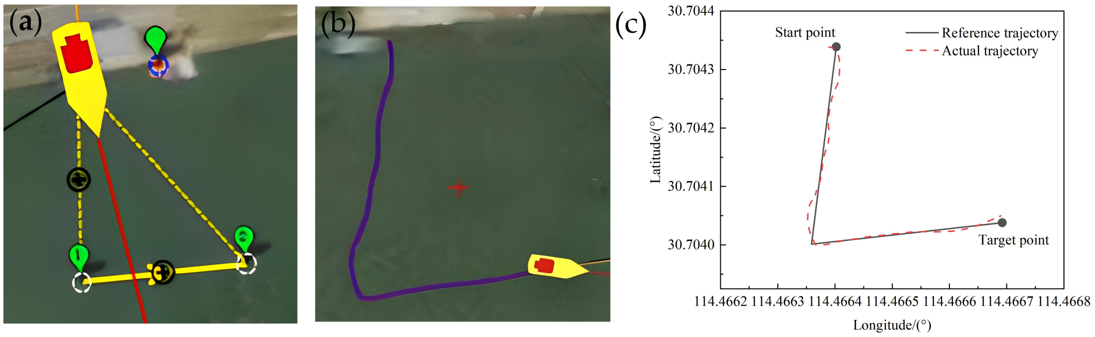

Fig 11. Trajectory of a triangle with an approximate 60° turning angle: (a) Reference trajectory; (b) Actual trajectory; (c) Comparison of the reference trajectory and the actual trajectory.

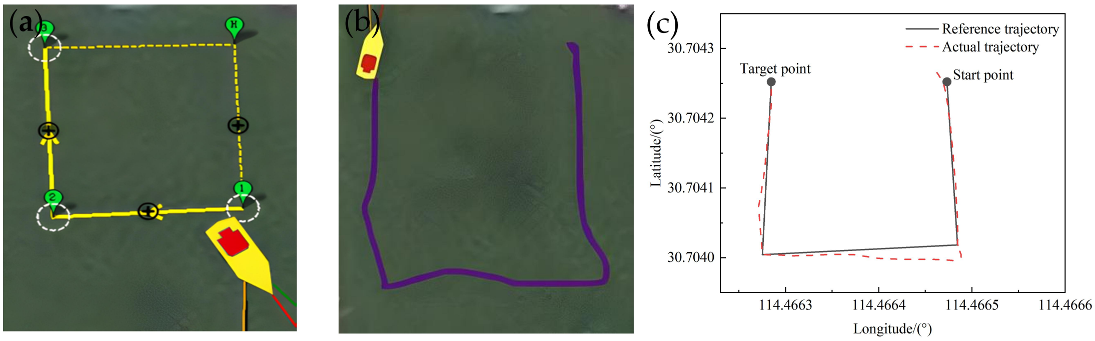

Fig 12. Trajectory of a square with an approximate 90° turning angle: (a) Reference trajectory; (b) Actual trajectory; (c) Comparison of the reference trajectory and the actual trajectory.

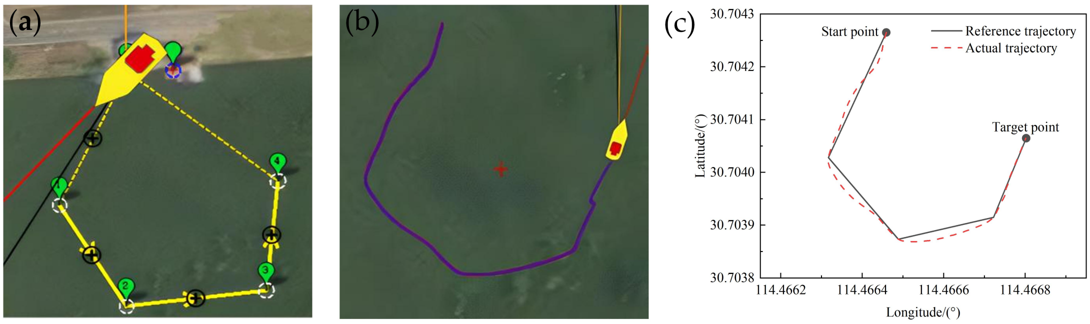

Fig 13. Trajectory of a pentagon with an approximate 120° turning angle: (a) Reference trajectory; (b) Actual trajectory; (c) Comparison of the reference trajectory and the actual trajectory.
